# Early Antipsychotic Treatment in Juvenile Rats Elicits Long-Term Alterations to the Dopamine Neurotransmitter System

**DOI:** 10.3390/ijms17111944

**Published:** 2016-11-22

**Authors:** Michael De Santis, Jiamei Lian, Xu-Feng Huang, Chao Deng

**Affiliations:** 1 Antipsychotic Research Laboratory, Illawarra Health and Medical Research Institute, Wollongong, NSW 2522, Australia; mds953@uowmail.edu.au (M.D.S.); jl841@uowmail.edu.au (J.L.); 2 School of Medicine, University of Wollongong, Wollongong, NSW 2522, Australia; xhuang@uow.edu.au

**Keywords:** antipsychotic, dopamine, risperidone, olanzapine, aripiprazole, development, juvenile

## Abstract

Prescription of antipsychotic drugs (APDs) to children has substantially increased in recent years. Whilst current investigations into potential long-term effects have uncovered some alterations to adult behaviours, further investigations into potential changes to neurotransmitter systems are required. The current study investigated potential long-term changes to the adult dopamine (DA) system following aripiprazole, olanzapine and risperidone treatment in female and male juvenile rats. Levels of tyrosine hydroxylase (TH), phosphorylated-TH (p-TH), dopamine active transporter (DAT), and D_1_ and D_2_ receptors were measured via Western blot and/or receptor autoradiography. Aripiprazole decreased TH and D_1_ receptor levels in the ventral tegmental area (VTA) and p-TH levels in the prefrontal cortex (PFC) of females, whilst TH levels decreased in the PFC of males. Olanzapine decreased PFC p-TH levels and increased D_2_ receptor expression in the PFC and nucleus accumbens (NAc) in females only. Additionally, risperidone treatment increased D_1_ receptor levels in the hippocampus of females, whilst, in males, p-TH levels increased in the PFC and hippocampus, D_1_ receptor expression decreased in the NAc, and DAT levels decreased in the caudate putamen (CPu), and elevated in the VTA. These results suggest that early treatment with various APDs can cause different long-term alterations in the adult brain, across both treatment groups and genders.

## 1. Introduction

Prescription and use of antipsychotic drugs (APDs) in children and adolescents is increasing rapidly worldwide, despite a lack of knowledge on the safety and efficacy of APD use on the developing brain [1,2,3,4,5,6,7,8,9]. Second-generation APDs including aripiprazole, olanzapine and risperidone are commonly being prescribed (mostly off-label) for the treatment of a variety of childhood disorders, from mental illnesses including anxiety, depression and child-onset schizophrenia [5,10], to various behavioural disorders [11,12,13]. Risperidone especially has been found to be highly effective in the treatment of male childhood behavioural disorders, and subsequently approved for use by regulatory and governing bodies, and prescribed at a higher rate [12]. Furthermore, recent investigations have also highlighted the potential capability of APD treatment in reducing suicidal risk factors, commonly associated with the aforementioned mental illnesses [14].

Although APDs are known to produce their therapeutic effects predominantly through potent antagonistic and/or partial agonist mechanisms of action on the dopamine (DA) D_2_ and serotonin (5-HT) 5-HT_1A_ and 5-HT_2A/2C_ receptors [15,16,17,18,19,20,21], the dopaminergic and serotonergic neurotransmitter (NT) systems also undergo, and are heavily involved in, multiple critical neurodevelopmental processes during the childhood/adolescent period [18,22,23,24,25,26,27].

There is therefore the potential that use of potent APDs at this critical time period of neurodevelopment has the ability to cause long-term alterations to NT systems, including DA signalling pathways, in a manner preceding normal brain functioning [8,26,28]. With alterations to mesocortical, mesolimbic and nigrostriatal DA NT pathways previously implicated in the pathophysiology of the mental illness state [15,21,29,30], prescription and use of APDs in the childhood/adolescent period may be potentially leading to long-term deficits in brain functioning [31].

Whilst current clinical investigations into the effects of APD use in the adolescent population has found some benefits in the control of various mental illness symptomology short term (1–2 months) and over a longer period (up to six months) [32,33,34], whether or not childhood/adolescent APD use is causing long-lasting alterations to adult brain functioning is mostly unknown [18,35,36].

Previously, several animal studies investigating the effects of early APD use on the developing brain, including previous investigations completed in our laboratory, has found that early treatment of up to 4 weeks can result in various significant alterations to both behavioural attributes [31], and numerous NT systems, including the DAergic NT system [18,36,37,38,39,40]. Whilst numerous investigations have found that early treatment with various APDs in juvenile rats has resulted in various short-term alterations immediately after treatment [36,38,40], studies into potential long-term alterations are fewer in number [18,37]. Changes to both the distribution/density of various NT receptor subtypes, including DA D_1_ and D_2_ receptors have been uncovered in cortical and striatal brain regions, along with alterations to dendritic architecture [41], with limited evidence on potential long-lasting effects to the synthesis, production and regulation of DA.

The aim of the current study was therefore to further investigate the long-lasting effects of early APD exposure in juveniles with aripiprazole, olanzapine and risperidone on the DA NT system in both male and female adult rats. In particular, adult brain levels of dopamine active transporter (DAT), and the DA synthesis markers tyrosine hydroxylase (TH) and phosphorylated-tyrosine hydroxylase (p-TH), as well as D_1_ and D_2_ receptors, were investigated in both cortical and striatal brain regions, along with regions involved in the synthesis and regulation of the DA signal, including the hippocampus, substantia nigra (SN) and ventral tegmental area (VTA), via Western blot and/or receptor autoradiography experiments.

## 2. Results

### 2.1. Long-Term Effects of Adolescent APD Treatment on Tyrosine Hydroxylase (TH) and Phosphorylated-Tyrosine Hydroxylase (p-TH) Levels

#### 2.1.1. Tyrosine Hydroxylase

**Figure 1:**
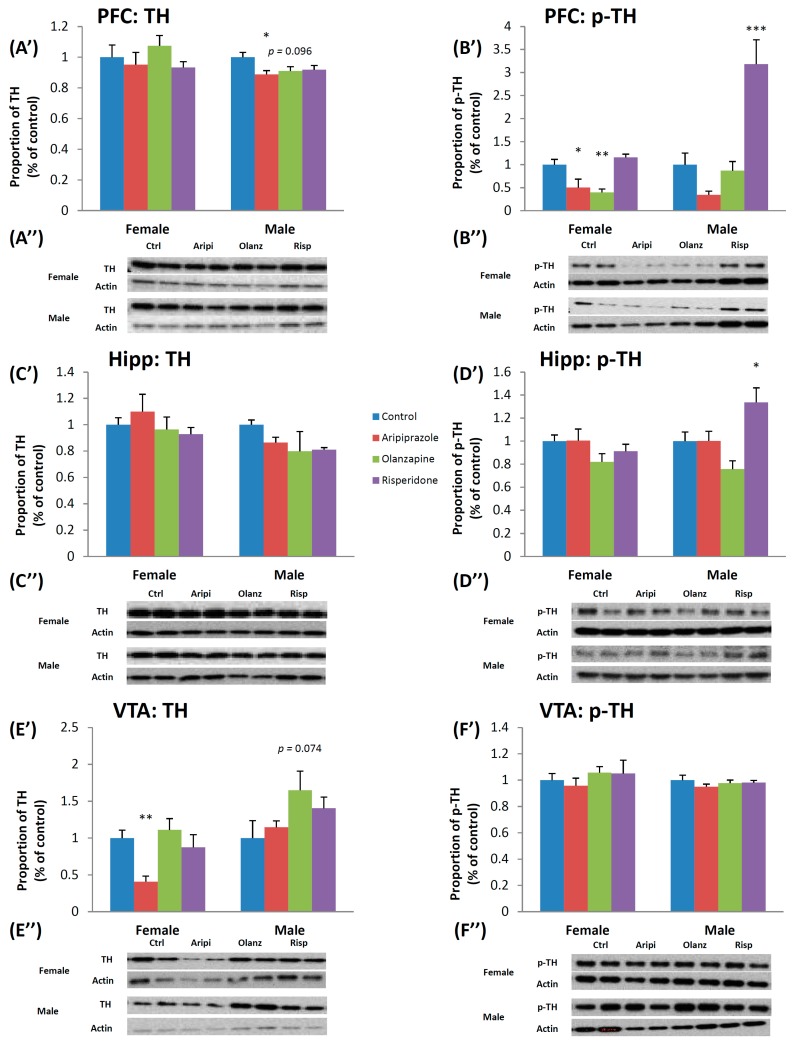
Effects of three APDs on TH and p-TH expression levels in the PFC (TH: (**A’**,**A”**); p-TH: (**B’**,**B”**)), Hipp (TH: (**C’**,**C”**); p-TH: (**D’**,**D”**)) and VTA (TH: (**E’**,**E”**); p-TH: (**F’**,**F”**)) of female and male rats. Sprague-Dawley rats were treated chronically with Aripiprazole (1.0 mg/kg, t.i.d), Olanzapine (1.0 mg/kg, t.i.d), Risperidone (0.3 mg/kg, t.i.d) or control (vehicle). Data expressed as mean ± SEM. * *p* < 0.05, ** *p* < 0.01, *** *p* < 0.001 vs. control. The representative bands of Western Blot are shown. APD: Antipsychotic drug; Hipp: Hippocampus; p-TH: Phosphorylated-tyrosine hydroxylase; PFC: Prefrontal cortex; t.i.d.: Three times daily; TH: Tyrosine hydroxylase; VTA: Ventral Tegmental Area.

There was a significant effect of Gender on TH expression in the nucleus accumbens (NAc) (*F*_1,46_ = 10.487, *p* < 0.01), SN (*F*_1,44_ = 38.456, *p* < 0.001), Hippocampus (*F*_1,45_ = 5.416, *p* < 0.05) and VTA (*F*_1,46_ = 15.486, *p* < 0.001) following analysis via two-way ANOVA (Analysis of Variance). Post hoc analysis revealed that whilst males expressed higher TH density levels in the SN, Hippocampus and VTA, females were found to have higher levels in the NAc. Furthermore, a significant effect of treatment on TH expression was found in the VTA (*F*_3,46_ = 4.851, *p* < 0.01) (Figure 1E’,E”), whilst a trend to significant effect of Treatment was observed in the caudate putamen (CPu) (*F*_3,46_ = 2.352, *p* = 0.087). Analysis via one-way ANOVA of the female cohort uncovered a significant effect of early APD treatment on TH expression in the VTA (*F*_3,22_ = 6.098, *p* < 0.01). Further post hoc tests found a significant decrease in TH expression following early treatment with the APD aripiprazole (−59.2%, *p* < 0.01). In the male cohort, a significant effect of early APD treatment on TH expression was found in the prefrontal cortex (PFC) (*F*_3,22_ = 3.201, *p* < 0.05) of adult rats. Post hoc analysis discovered that early aripiprazole treatment significantly decreased TH expression (−11.2%, *p* < 0.05), whilst a trend to significant decrease was also found in the olanzapine drug treatment group (−9.0%, *p* = 0.096) (Figure 1A’,A”).

#### 2.1.2. Phosphorylated-Tyrosine Hydroxylase

Analysis via two-way ANOVAs (Gender × Treatment) of p-TH expression levels revealed a significant effect of Gender (*F*_1,46_ = 11.602, *p* < 0.01), Treatment (*F*_3,46_ = 6.759, *p* < 0.001) and a significant interaction between the two factors (*F*_3,46_ = 8.272, *p* < 0.001) in the PFC of animals, with a higher expression in the male cohort. In the SN, a significant effect of Gender was also observed (*F*_1,44_ = 6.884, *p* < 0.02), with higher p-TH expression in the female cohort, whilst in the Hippocampus, a significant effect of Treatment (*F*_3,45_ = 5.301, *p* < 0.01) and a significant interaction between the two factors (*F*_3,45_ = 3.430, *p* < 0.05) was uncovered. In the female cohort, analysis via one-way ANOVA uncovered a significant effect of early APD treatment on p-TH expression in the PFC (*F*_3,22_ = 9.070, *p* < 0.01), whilst post hoc analysis revealed p-TH expression in the PFC was found to significantly decrease in both the aripiprazole (−49.4%, *p* < 0.05) and olanzapine (−60.2%, *p* < 0.01) treatment groups (Figure 1B’,B”). In the male cohort, analysis via one-way ANOVA found a significant effect of treatment on p-TH expression in the PFC (*F*_3,22_ = 15.887, *p* < 0.001) and Hippocampus (*F*_3,22_ = 6.103, *p* < 0.01). Post hoc analysis revealed that early risperidone treatment significantly increased p-TH expression in both the PFC (218.3%, *p* < 0.001) (Figure 1B’,B”) and Hippocampus (33.7%, *p* < 0.05) (Figure 1D’,D”) of male rats.

### 2.2. Long-Term Effects of Adolescent APD Treatment on Dopamine Active Transporter (DAT) Levels

Two-way ANOVA analysis revealed a significant effect of Gender found in the CPu (*F*_1,46_ = 6.509, *p* < 0.02), NAc (*F*_1,44_ = 6.300, *p* < 0.02) and VTA (*F*_1,46_ = 15.977, *p* < 0.001) of adult rats following early APD treatment, with a higher protein expression of DAT in the male cohort uncovered. Furthermore, a trend to significant interaction between the Gender and Treatment factors was found in the VTA (*F*_3,46_ = 2.554, *p* = 0.07). One-way ANOVA analysis of the male cohort found that early APD treatment had a significant effect on DAT protein expression in the VTA (*F*_3,22_ = 3.170, *p* < 0.05). Furthermore, post hoc analysis revealed that risperidone treatment significantly increased DAT protein expression in the VTA (+107.3%, *p* < 0.05). No significant differences in DAT protein expression were found following analysis of the female cohort.

**Figure 2:**
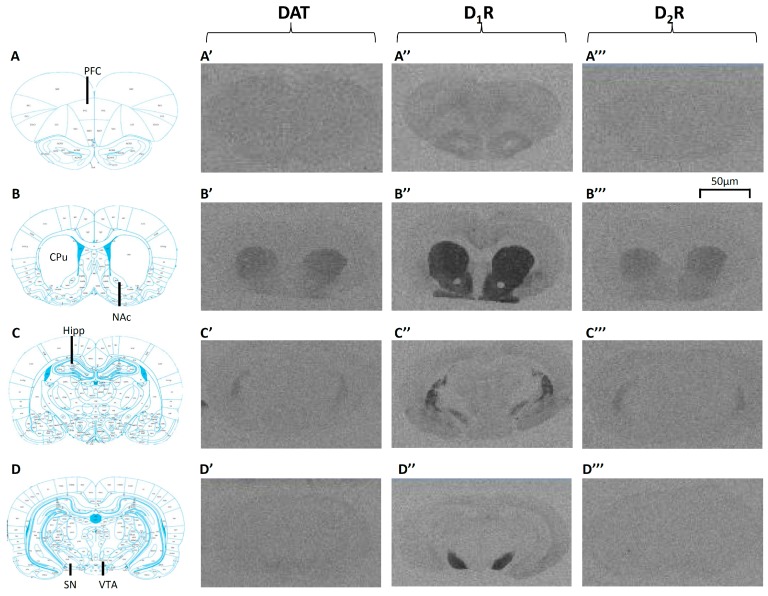
Examples of DAT, D_1_ and D_2_ receptors binding in the adult rat brain following childhood/adolescent APD treatment. (**A**–**D**) The schematic diagram adapted from a rat brain atlas [42] showing the level of Bregma for each investigated region ((**A**) PFC: 4.68 mm; (**B**) CPu & NAc: 1.08 mm; (**C**) Hippocampus: −2.76 mm; (**D**) SN & VTA: −4.92 mm). (**A’**–**D’**) Examples of autoradiograms to demonstrate [^3^H]WIN35428 binding to DAT; (**A”**–**D”**) Examples of autoradiograms to demonstrate [^3^H]SCH23390 binding to DA D_1_ receptors (D_1_R); (**A’’’**–**D’’’**) Examples of autoradiograms to demonstrate [^3^H]Raclopride to DA D_2_ receptors (D_2_R). APD: Antipsychotic drug; CPu: Caudate Putamen; DA: Dopamine; DAT: Dopamine Active Transporter; Hipp: Hippocampus; NAc: Nucleus Accumbens; PFC: Prefrontal cortex; SN: Substantia Nigra; VTA: Ventral Tegmental Area.

Examples of [^3^H]WIN35428 binding to DAT are presented in Figure 2A’–D’. Non-specific binding was observed to be less than 20% in the CPu, and less than 25% in the SN and VTA due to relatively lower DAT binding densities. There was a significant effect of Gender on DAT bindings in the SN (*F*_1,44_ = 11.803, *p* < 0.01) and VTA (*F*_1,46_ = 13.941, *p* < 0.01) of rats following two-way ANOVA, with significantly higher levels of DAT bindings in the male cohort. Furthermore, a significant effect of Treatment was found in the CPu (*F*_3,46_ = 2.976, *p* < 0.05). Post hoc analysis revealed that early risperidone treatment significantly decreased DAT binding by −35.9% (*p* < 0.02). Arranged by gender, one-way ANOVA revealed a significant effect of early APD treatment on DAT bindings was observed in the CPu of the male cohort (*F*_3,21_ = 4.065, *p* < 0.05). Post hoc analysis revealed that early risperidone treatment significantly decreased DAT bindings in the CPu (−37.1%, Controls 176.9 ± 8.4 fmoles/mg vs. risperidone 111.3 ± 15.7 fmoles/mg, *p* < 0.02). Due to very weak DAT binding present in the PFC, NAc and Hippocampus of both females and males, DAT bindings in these areas were not quantified. No significant effects were found in the female cohort between APD treatment group and controls.

### 2.3. Long-Term Effects of Adolescent APD Treatment on Dopamine D_1_ Receptor (D_1_R) Levels

**Figure 3:**
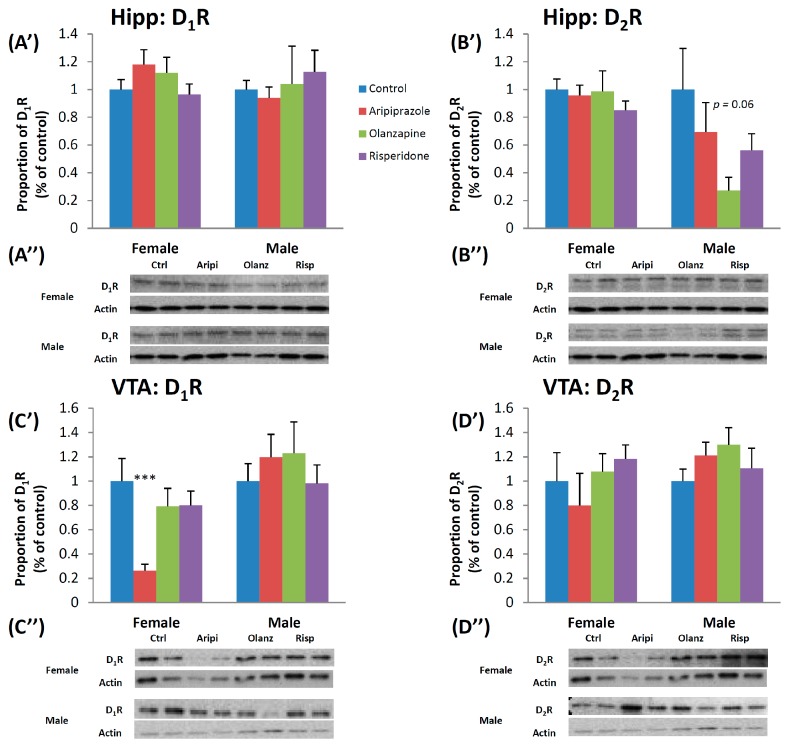
Effects of three APDs on D_1_ and D_2_ receptor expression levels in the Hippocampus (D_1_R: (**A’**,**A”**); D_2_R: (**B’**,**B”**)) and VTA (D_1_: (**C’**,**C”**); D_2_: (**D’**,**D”**)) of female and male rats. Sprague-Dawley rats were treated chronically with Aripiprazole (1.0 mg/kg, t.i.d), Olanzapine (1.0 mg/kg, t.i.d), Risperidone (0.3 mg/kg, t.i.d) or control (vehicle). Data expressed as mean ± SEM. *** *p* < 0.001 vs. control. The representative bands of Western blot are shown. APD: Antipsychotic drug; Hipp: Hippocampus; t.i.d.: Three times daily; VTA: Ventral Tegmental Area.

**Figure 4:**
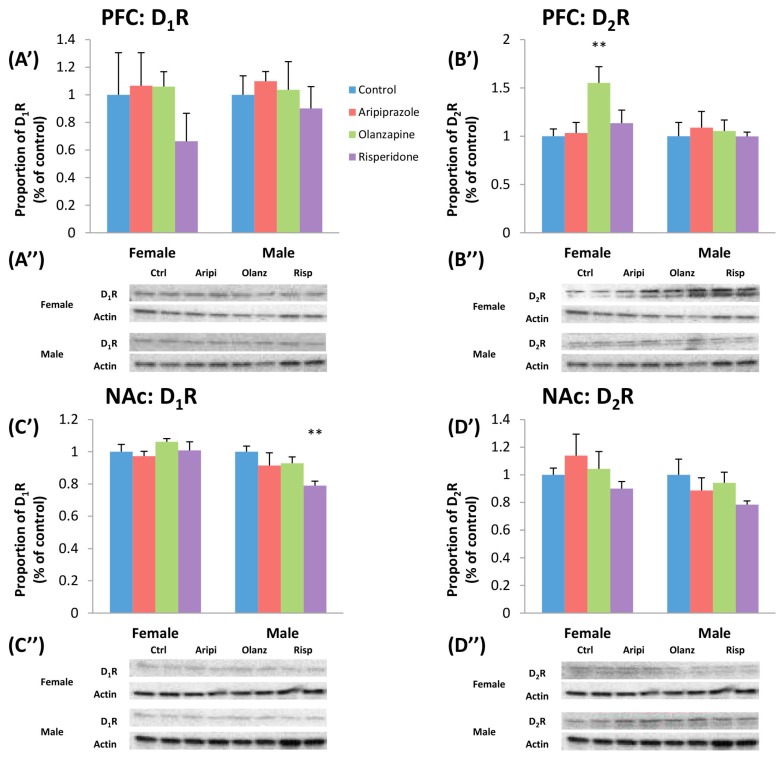
Effects of three APDs on D_1_ and D_2_ receptor (D_1_R and D_2_R) expression levels in the PFC (D_1_R: (**A’**,**A”**); D_2_R: (**B’**,**B”**)) and NAc (D_1_R: (**C’**,**C”**); D_2_R: (**D’**,**D”**)) of female and male rats. Sprague-Dawley rats were treated chronically with Aripiprazole (1.0 mg/kg, t.i.d), Olanzapine (1.0 mg/kg, t.i.d), Risperidone (0.3 mg/kg, t.i.d) or control (vehicle). Data expressed as mean ± SEM. ** *p* < 0.01 vs. control. The representative bands of Western blot are shown. APD: Antipsychotic drug; NAc: Nucleus Accumbens; PFC: Prefrontal cortex; t.i.d.: Three times daily.

Two-way ANOVA found a significant effect of Gender on the protein levels of D_1_R in the NAc (*F*_1,45_ = 11.529, *p* < 0.01), with a higher D_1_ receptor protein level in the female cohort, whilst male protein levels of D_1_R was found to be higher overall in the VTA (*F*_1,46_ = 1.720, *p* < 0.01). A significant interaction between Gender and Treatment factors in the VTA (*F*_3,46_ = 3.273, *p* < 0.05) was also uncovered. In the female cohort, one-way ANOVA uncovered a significant long-lasting effect of treatment observed in the VTA (*F*_3,22_ = 5.551, *p* < 0.01). Post hoc analysis revealed that early aripiprazole treatment significantly decreased D_1_R protein levels in the VTA by 73.7% (*p* < 0.02) (Figure 3C’,C”). Analysis of the male cohort revealed early APD treatment had significant long-term effects on the D_1_R protein levels in the NAc (*F*_3,21_ = 3.749, *p* < 0.05). Post hoc tests demonstrated that early treatment with risperidone was found to decrease D_1_R protein levels by 21.0% (*p* < 0.02) (Figure 4C’,C”).

Examples of [^3^H]SCH23390 binding to D_1_R are presented in Figure 2A”–D”. DA D_1_ receptor binding was observed in the CPu, NAc and SN, with non-specific binding was observed to be less than 10%. However, there was a weak binding in the PFC, Hippocampus and VTA, and was therefore discounted from analysis. There was a significant effect of Gender in the SN (*F*_1,46_ = 20.093, *p* < 0.001) of rats following two-way ANOVA, with higher levels uncovered in the female cohort. Additionally, a trend to a significantly higher D_1_R binding was found in the CPu of female rats (*F*_1,46_ = 2.966, *p* = 0.093).

### 2.4. Long-Term Effects of Adolescent APD Treatment on Dopamine D_2_ Receptor (D_2_R) Levels

There was a significant effect of Gender on DA D_2_R protein levels in the SN (*F*_1,45_ = 35.633, *p* < 0.001) and hippocampus (*F*_1,45_ = 7.418, *p* < 0.02) following two-way ANOVA, with higher D_2_R protein levels found in the female cohort in both brain regions. In the female cohort, one-way ANOVA analysis revealed that APD treatment had a significant effect on D_2_R protein in the PFC (*F*_3,21_ = 4.228, *p* < 0.02). Early treatment with olanzapine significantly increased D_2_R protein in the PFC by 55.1% (*p* < 0.02) (Figure 4B’,B”). Whilst no significant alterations to D_2_R protein levels were found between drug treatment groups in the male cohort, a trend to significant effect of treatment was found in the SN (*F*_3,21_ = 2.897, *p* = 0.064) and in the hippocampus between olanzapine and control groups (−72.8%, *p* = 0.06) (Figure 3B’,B”).

Examples of [^3^H]Raclopride binding to D_2_R are presented in Figure 2A’’’–D’’’. Non-specific binding in the CPu and NAc were observed to be below 10%. Weak binding, however, was found in the PFC, Hippocampus, SN and VTA, and were thus discounted from analysis. Analysis via two-way ANOVAs (Gender × Treatment) uncovered a trend towards a significant interaction between the two factors on D_2_R binding density in the NAc (*F*_1,46_ = 3.790, *p* = 0.059). Analysis of the female cohort via one-way ANOVA, a significant effect of treatment on D_2_R expression was found in the NAc (*F*_3,24_ = 3.362, *p* < 0.05). Post hoc analysis demonstrated that early olanzapine treatment significantly increased D_2_R binding in the NAc of female rats (+35.0%, Control 79.8 ± 7.5 fmoles/mg vs. olanzapine 107.6 ± 6.8 fmoles/mg, *p* < 0.05), whilst trends to significant increases were found following early treatment of olanzapine (+11.8%, Control 161.2 ± 1.5 fmoles/mg vs. olanzapine 180.2 ± 9.0 fmoles/mg, *p* = 0.083) in the CPu of adult female rat brains. No significant alterations were found following analysis in the male cohort.

## 3. Discussion

The present study investigated for the first time the long-term effects of early treatment (in juvenile rats) with the commonly used APDs aripiprazole, olanzapine and risperidone, on DA neurotransmission in the hippocampus, SN and VTA, and provides further evidence of potential alterations in the PFC, CPu and NAc in adult male and female rats. Our findings provide evidence that early APD treatment during the critical neurodevelopmental period of youth causes long-lasting alterations to DA synthesis and re-uptake markers in the mesocortical DA NT pathway, CPu and Hippocampus in the adult brain. Additionally, alterations to DA D_1_ and D_2_ receptors were also uncovered across the mesolimbic and nigrostriatal brain regions. Furthermore, different effects between gender cohorts were also uncovered.

Whilst previous studies investigating the long-term effect of juvenile APD use on DA synthesis markers in the adult brain have to our knowledge not been completed, several studies investigating the immediate and long-lasting effects of APD treatment with aripiprazole, olanzapine and risperidone on DA synthesis markers, precursors, and NT levels in young [37,43] and adult [43,44,45,46,47,48,49,50] rodent models have demonstrated differing effects.

In this study, long-term alterations in DA synthesis markers were uncovered in the present study. In the female cohort, significant decreases in the DA synthesis markers TH and p-TH were found following early treatment with aripiprazole and olanzapine in comparison to the control; specifically in the PFC and VTA brain regions of the DA NT system. Rats that received early treatment with aripiprazole showed decreased levels of TH and p-TH in the VTA and PFC respectively, whilst early olanzapine treatment resulted in decreased p-TH levels in the PFC upon comparison to the control. Additionally, decreased TH levels in the PFC were also uncovered in the male cohort following early aripiprazole and olanzapine treatment. Previous investigations into the effect of the partial D_2_ agonist aripiprazole over acute and short-term durations in the adult rodent have uncovered increases to DA markers indicative of an increased DA synthesis and/or production in the PFC of male rats [43,44,45,46,47]. However, studies into the effects of short-term aripiprazole treatment on DA production in the VTA have revealed an opposite effect, with reductions in DA firing observed [48,50]. Furthermore, whilst investigations into the effects of olanzapine over short-term time periods in adult rats have also found increases to DA and its precursor 3,4-dihydroxyphenylalanine (DOPA) in the PFC of treated animals [44], an investigation into the long-term effects of adolescent treatment with olanzapine on the stimulated release of the DA NT in the adult brain revealed decreases to DA transmission in the NAc following electrical stimulation of the VTA [37].

Interestingly, our study revealed that early treatment with risperidone, however, was found to increase p-TH levels in both the PFC and hippocampus of male rats. Previous acute and short-term studies into the effects of the potent D_2_ receptor antagonist risperidone revealed increased levels of DA production and transmission in the PFC and NAc of the adult rats [49,51]. This indicates that whilst the actions of all APDs in the juvenile neurodevelopmental phases resulted in long-term alterations to DA synthesis levels, the more potent D_2_ antagonist pharmacology of risperidone resulted in an increase to the level of DA synthesis markers in the long-term, compared with the decreased levels observed in the other APDs investigated. Furthermore, a clear difference between genders was observed with the change elicited by risperidone only observed in the male rodent model.

Whilst similar effects of aripiprazole treatment on DA production in the VTA, and risperidone treatment effects on DA levels in the PFC was observed in the present study, opposing effects to those previously described in the literature were also revealed. Specifically, decreases in the production of TH and p-TH were found in the PFC of both female and male animals following treatment with both aripiprazole and olanzapine in our study. It is possible that these opposing results to that reported in the literature may be due to factors including; the difference in age of the animals treated, the treatment duration of the study, and the duration of time between cessation of treatment and detection of DA markers, with the current investigation specifically investigating what the effects of early APD treatment is on DA NT synthesis in the adult brain. The observed alterations to the measured variables may have therefore subsequently occurred following the withdrawal period following the cessation of APD treatment. Furthermore, the altered levels of TH and p-TH observed following aripiprazole, olanzapine and risperidone treatment may have been the result of its pharmacological actions on DA receptors undergoing a critical neurodevelopmental period, which has previously been found to result in long-term alterations to NT functioning [18,37].

Although investigations into the long-term effects of early APD treatment on DAT levels have not been completed in either young or adult treatment models to our knowledge, adult animal studies demonstrating the acute and short-term effects of drugs with a pharmacology based around the DA D_2_ receptor on DAT availability and subsequent DA re-uptake have shown region specific results [52,53,54]. Along with alterations in DA synthesis markers, our study uncovered region specific alterations to DAT levels in the male cohort. Whilst early APD treatment with risperidone was found to increase DAT expression in the VTA (in correlation to the effect seen on TH and p-TH levels in the PFC and hippocampus), a decrease in DAT level was observed in the CPu in comparison to the control. Previous studies into the effects of D_2_ receptor antagonists have found decreases to DAT re-uptake and availability in striatal regions, whilst opposite effects have been observed in the NAc [52,54]. Additionally, investigations into the effects of D_2_ receptor agonists have indeed uncovered an increase in DAT availability in the striatum [52,54].

As previously mentioned, whilst the observed alterations to DAT levels were parallel to the majority of changes in DA synthesis markers, the decrease in DAT expression in the CPu of the male cohort following risperidone treatment was not expected. This potentially indicates that whilst APD treatment may result in an up/down-regulation of DA synthesis, and concurrent alteration in DAT in mesocortical/mesolimbic DA pathways, different alterations may be caused along other DAergic projections. With alterations to specific DAergic regions/projections previously implicated in numerous facets of mental illness symptomology, including alterations to activity levels [17,55] and cognitive functioning [56], such alterations following early APD treatment may be indicative of changes to such symptomology.

With the pharmacology of the APDs investigated in our study based around either partial agonist or antagonist mechanism of action of the DA D_2_ receptor, it can be said that strong similarities can be drawn between results. Whilst it is clear that the levels of DAT present in the investigated DA pathways are heavily influenced by the pharmacological actions of drugs on the DA D_2_ receptor, it is also clear that different effects of APD treatment may also be observed between brain regions [53]. Similar region specific alterations were also observed in our investigation, and it has been heavily postulated that this may be due to the differences in the density and availability of DA receptors (both D_1_ and D_2_) between brain regions, and the subsequent ability of agonist/antagonist actions on these receptors to influence levels of DAT.

Regional differences in adult DA D_1_ receptor densities were also uncovered following juvenile APD treatment in both the female and male cohorts. In the female cohort, a decreased D_1_ receptor density was found in the VTA of female animals treated with the partial agonist aripiprazole, whilst a similar decrease in D_1_ receptor levels were found in the male cohort, with early risperidone treatment found to cause long-term decreases to D_1_ receptor density in the NAc. Whilst similar investigations into the adult animal model has found no alterations to DA D_1_ receptor density levels in the PFC, CPu, NAc and hippocampus following long-term olanzapine and risperidone drug treatment [57], similar decreases in DA D_1_ receptor levels have been uncovered in previous investigations in young animals. Studies investigating the immediate effects of juvenile APD treatment following a short-term treatment period [38,40], and long-lasting effects following long-term treatment period [18,37], has found that both olanzapine and clozapine resulted in a decreased D_1_ receptor density level in the PFC and NAc of young male rats.

Physiological differences between genders, variances in the density and availability of DA D_1_ receptors across the investigated brain regions, along with the pharmacological actions of the APDs used may account for the region and treatment factor differences in DA D_1_ receptor density levels observed. More specifically, with the D_1_ receptors playing a critical role in the regulation of DA transmission along DAergic projections via facilitation from hippocampal regions [15,58,59], the partial agonist actions of aripiprazole on the D_2_ receptor pre-synaptically will subsequently be heavily influenced by the concentration of extracellular DA NT linked to D_1_ receptor density. This potentially may result in the observed alterations in the present study, with decreased D_1_ receptor density uncovered in the VTA of female rats following aripiprazole treatment, whilst risperidone was found to increase D_1_ receptor density in hippocampal regions.

The present study found increases in the expression of the DA D_2_ receptor in numerous brain regions in the female rodent model between drug treatment group and control. Female rats that received early olanzapine drug treatment exhibited increased D_2_ receptor binding density in the PFC, NAc and CPu when compared to the control group. Previous investigations into the effects of APD treatment on DA D_2_ receptor levels in an animal model have also uncovered either similar significant increases to D_2_ receptor expression following treatment, or no change to D_2_ receptor expression across both male and female cohorts [36,37,38,40,43,57,60,61,62]. Whilst studies into the effects of both short and long-term APD treatment with olanzapine and risperidone on D_2_ receptor densities have found increases in regions including the PFC, CPu, NAc and hippocampus, both immediately following cessation of treatment [38,57] and after long-term time periods [18,37,38,57], the effects of treatment with the partial agonist drug aripiprazole, over short and long-term treatment periods, have found conflicting results. APD treatment in both young and adult rat models have been found to result in no immediate alterations to D_2_ receptor density in striatal brain regions [43,60,61], whilst a more recent investigation in young rats uncovered an increased expression of D_2_ receptor levels in the CPu following a short-term treatment period [36]. Surprisingly, despite the well-document high affinity and mechanism of action of APDs through DA D_2_ receptors, no alterations to D_2_ receptor levels were observed in the male cohort in any of the investigated regions in the present study.

Whilst the current study investigated the effects on the D_1_ and D_2_ receptors specifically due to their high expression levels in investigated regions, there is also the potential for juvenile APD treatment to cause long-term alterations to DA D_3_ receptor levels in the adult brain. With the D_3_ receptor linked to both the therapeutic effects of APD actions, and correlated to the expression of D_2_ receptors [63], further investigations into potential impacts of juvenile APD expression will shed further light on the depth of the effects of early APD treatment on the DA NT system. Additionally, it is well documented that in the majority of cases, a multi-drug approach is implemented in the clinic [64], and there is subsequently the potential for a multi-drug treatment approach to result in similar or even more widespread alterations. Therefore, further studies into the effects of APD co-treatment with other drugs (such as the antidepressant bupropion) have the potential to uncover further detrimental effects of these drugs on the developing brain.

The current study has uncovered numerous gender-specific effects across multiple measured variables as a result of juvenile APD treatment. Most profoundly however, were the observed opposing effects of APD treatment on p-TH and DA D_2_ receptor levels, upon comparison of the male and female cohorts. As mentioned in a previous article, there are numerous potential factors that may be resulting in the observed variances between genders [31]. Firstly, the well-documented gender difference in neurodevelopmental phases of the DA system has the potential to have an obvious significant influence on the observed differences in the present study. In particular, previous investigations have uncovered both a regional and gender-based difference in the development of the DA NT system, including differences in; striatal DA D_1_ and D_2_ receptor overproduction and elimination, DA D_1_ receptor densities in the NAc, and rates of myelination [25,55]. Together, these gender-based differences in the neurodevelopmental phases of the DA NT system in particular, combined with the highly potent pharmacological mechanism of action of APDs in the critical neurodevelopmental window, may explain the observed differences in DA-related measured variables. Furthermore, with such long-term differences in APD treatment response to measured DA variables across genders observed in this study, there is also subsequently the potential gender differences in clinical response to prescribed APD treatments later in life.

Additionally, the sex hormones testosterone and oestrogen may also have played a significant role in the observed gender differences of the present study, in particular the dissimilar alterations to p-TH expression following APD treatment. It is well documented that during adolescence, both testosterone and oestrogen strongly influence the development and maturation of the brain in DAergic regions, including the PFC and striatum [17,25,55]. Similarly, they are also known to play a significant role in shaping the DA signal, with previous animal studies that augmented both testosterone and oestrogen levels found correlative alterations to DA neurotransmission, including DA synthesis, receptor mRNA and transporter levels [17,55,65]. Furthermore, studies have also uncovered some “neuro-protective” effects of oestrogen on the DA NT system, with the hormone exhibiting an ability to inhibit DA D_2_ receptor, along with 5-HT_1A_ receptor-induced mediated behavioural changes in sensorimotor gating/information processing, of which has previously been found deficient in people with mental illnesses [66,67].

## 4. Materials and Methods

### 4.1. Animals and Housing

Timed pregnant Sprague-Dawley rats were obtained at gestation day 16 from the Animal Resource Centre (Perth, Australia). They were housed in individual cages under environmentally controlled conditions (22 °C, light cycle from 07:00 to 19:00 and dark cycle from 19:00 to 07:00), and allowed ad libitum access to water and standard laboratory chow diet (3.9 kcal/g: 10% fat, 74% carbohydrate, 16% protein). Day of birth was considered postnatal day (PD) 0. Pups were sexed on PD14, and then 96 Sprague-Dawley rats (48 males and 48 females) were weaned on PD20 and housed in individual cages.

### 4.2. Drug Treatment Groups

Before the drug treatment commenced, the rats were trained for self-administration by feeding them cookie dough (0.3 g) without drugs 2 times per day for PD 18–21. Animals were then assigned randomly to one of four experimental groups per gender on PD21 (*n* = 12/group): (1) aripiprazole (Otsuka, Tokyo, Japan); (2) olanzapine (Eli Lilly, Indianapolis, IN, USA); (3) risperidone (Apotex, Toronto, ON, Canada); or (4) control (vehicle). The drug treatment period from PD 22–50 in juvenile rats was carried out at the equivalent time of the childhood/adolescent phase in humans [25]. A staggered drug treatment pattern, where lower APD dosages are slowly increased to a final dosage amount, was used to mimic a clinical setting [68]. The APD doses were initiated on PD22 at 0.2 mg/kg for aripiprazole; 0.25 mg/kg, 3 times per day for olanzapine; and 0.05 mg/kg, 3 times per day for risperidone, and then increased in 3 steps over the first 7 days of the 4 week treatment period to achieve a final dose on PD28 of 1 mg/kg, 3 times per day for aripiprazole; 1 mg/kg, 3 times per day for olanzapine; and 0.3 mg/kg, 3 times per day for risperidone. Rats were observed throughout the treatment to ensure that they completely consumed the cookie dough pellet. The rats in the control group received an equivalent pellet without the drug. In consideration of a shorter half-life of APDs in rats [69], APDs were administered 3 times per day (at 07:00, 14:00 and 22:00; with 8 ± 1 h intervals) in order to ensure consistently high concentrations and mirror the clinical scenario of oral administration once per day. The proposed dosages are within the recommended dosage ranges for the psychiatric treatment of paediatric patients, based on the body surface area formula for dosage translation between humans and rats in the FDA guideline for clinical trials [34,68,70,71]. The relevant human equivalent dose (HED) is therefore calculated by the formula: Animal dose (mg/kg) × Animal Km (6)/Human Child Km (25) × Body Weight (Km factor, body weight (kg) divided by body surface area (m^2^), is used to convert the mg/kg dose to a mg/m^2^ dose). Therefore, for an adolescent with an average weight of 40 kg, the utilized dosages for aripiprazole (1 mg/kg) and olanzapine (1 mg/kg) equals 9.6 mg, whilst risperidone (0.3 mg/kg) equates to a dosage of 2.88 mg, all within a clinically relevant range. It has been previously reported that, at these used dosages, aripiprazole treatment reaches above 90% DA D_2_ receptor occupancy rates in the rat brain [72], while olanzapine and risperidone reach 65%–80% DA D_2_ receptor occupancy [73,74]. These dosages have also been shown to be physiologically and behaviourally effective in our laboratory, with similar dosages seen to induce weight gain and changes in hypothalamic neuropeptide Y expression in adolescent rats [75], whilst alterations to both DA and 5-HT receptor binding has been reported in juvenile rats [40]. All experimental procedures were approved by the Animal Ethics Committee, University of Wollongong, Wollongong, Australia (AE 12/20), and complied with Australian Code of Practice for the Care and Use of Animals for Scientific Purpose (2004).

### 4.3. Histological Procedures

All rats were sacrificed via carbon dioxide asphyxiation on PD106 between 09:00 and 11:30 to minimise the potential circadian-induced variation of protein expression. Following euthanasia, the brain tissue was immediately removed, frozen in liquid nitrogen, and stored at −80 °C until analysis. Six brains from each drug treatment group (*n* = 12) were then randomly selected for Western blot analyses (outlined in Section 4.3.1 and Section 4.4), and the remaining six brains from each treatment group were then used for receptor autoradiography experiments (outlined in Section 4.3.2 and Section 4.5). The brain regions involved in both dopaminergic signalling and the therapeutic actions of APDs, including the PFC, CPu, NAc, hippocampus, SN and VTA, were dissected in order to detect DA receptor, transporter, and synthesis levels.

#### 4.3.1. Histology—Microdissection (Western Blot Analyses)

Brain microdissection puncture techniques were used to collect aforementioned selected brain regions following a standard procedure in our laboratory [76,77,78,79]. Briefly, 500 µm thick sections were cut at −14 °C using a cryostat (Leica CM1850, Leica Microsystems, Wetzler, Germany) and collected bilaterally using a microdissection puncher on glass slides.

#### 4.3.2. Histology—Receptor Autoradiography

Brains selected for receptor autoradiography were sectioned coronally at −18 °C into 14 µm using a cryostat (Leica CM1850, Leica Microsystems, Wetzler, Germany). Sections were then thaw mounted onto Poly-l-Lysine (Sigma-Aldrich, Castle Hill, NSW, Australia) coated glass slides and stored at −20 °C. A set of sections from each animal was stained with 0.5% cresyl violet solution (Nissl staining) and used to confirm identification of anatomical structures.

### 4.4. Western Blot Analyses

All brain tissue dissected from individual rats (outlined in Section 4.3.1) were homogenised in ice-cold homogenising buffer (9.8 mL NP-40 cell lysis buffer (Invitrogen, Camarillo, CA, USA), 100 µL β-Glycerophosphate (50 mM; Invitrogen), 33.3 µL PMSF (0.3 M; Sigma-Aldrich, St. Louis, MO, USA), and 100 µL Protease Inhibitor Cocktail (Sigma-Aldrich)). The samples were then centrifuged, and the supernatant solution was then collected and stored at −80 °C until required.

Total protein concentration was quantified spectrophotometrically via Bio-Rad DC Protein Assay (#500-0114, Bio-Rad, Hercules, CA, USA) at A750 nm. A range of sample protein concentrations was pre-tested in each region (2, 2.5, 4, 5, 6, 7.5, 8, and 10 µg). Ten micrograms of protein was selected for PFC, CPu and NAc regions, whilst 8 µg of protein was selected for Hippocampus, SN and VTA regions as it best fitted the linear range of signal detection for all tested antibodies. Homogenised brain samples containing the aforementioned µg concentration of protein were then firstly heated at 95 °C in the loading buffer (950 µL Laemmli buffer (Bio-Rad) and 50 µL β-mercaptoethanol (Sigma-Aldrich)) for 5 min to denature the protein, then placed on ice and centrifuged for 2 min at 4 °C. The samples were then loaded into Criterion™ TGX™ 4%–20% Precast Gels (Bio-Rad), and underwent electrophoresis in SDS-PAGE running buffer (100 mL 10× SDS-PAGE running buffer (Bio-Rad) and 900 mL distilled water) at 140 V for 70 min. Proteins on the gels were then transferred onto a polyvinylidene difluoride (PVDF) membrane (Bio-Rad) electrophoretically using the Bio-Rad Midi Format 1-D Electrophoresis Systems for 1 h at 100 V.; in ice-cold transfer buffer (150 mL 10× Tris/Glycine Buffer (Bio-Rad), 300 mL cold methanol and 1050 mL distilled water). In order to detect the proteins of interest, PVDF membranes were incubated in Tris-Buffered Saline-Tween (TBST) (Sigma-Aldrich) solution containing 5% Blotting Grade Blocker (Non-Fat Dry Milk Powder) (Bio-Rad) for 1 h at room temperature. Membranes were then incubated overnight with the primary antibody, including; D_1_R (1:5000; #ab20066, Abcam, Cambridge, United Kingdom), D_2_R (1:5000; #ab21218, Abcam, Cambridge, United Kingdom), DAT (1:1000; #SC-14002, Santa Cruz, Dallas, TX, USA), TH (1:2000; #AB9983, Millipore, Toronto, ON, Canada) and p-TH (1:10,000; #AB5935, Millipore, Toronto, ON, Canada), diluted in TBST buffer containing either 1% Non-Fat Dry Milk Powder (D_1_R, D_2_R, DAT) or 1% Bovine Serum Albumin (BSA) (TH, p-TH). Following primary antibody incubation, TBST was used to wash membranes (3 × 5 min), followed by a 1 h incubation with horseradish peroxidase (HRP)-conjugated goat anti-rabbit secondary antibody (D_1_R and D_2_R—1:5000, DAT—1:4000, TH—1:3333, p-TH—1:3333; Millipore, Temecula, CA, USA) at room temperature. Secondary antibodies were diluted in TBST buffer containing either 1% Non-Fat Dry Milk Powder (D_1_R, D_2_R and DAT) or 1% BSA (TH and p-TH). Three TBST washes then followed secondary antibody incubation, and proteins of interest were visualised using Classico Western horseradish peroxidase (HRP) Substrates (Millipore) and AmershamHyperfilm ECL (GE Healthcare, Life Science, Wauwatosa, WI, USA). Membranes were then re-probed with mouse anti-actin polyclonal antibody (1:10,000; Millipore, #MAB1501) and HRP-conjugated rabbit anti-mouse secondary antibody (1:3000; Cell Signalling, #7076). 

Immunoreactive signals were quantified using GS-800 image densitometry and Quantity One software (Bio-Rad, Version 4.6.7), and the values were corrected based on their corresponding actin levels. For D_1_R, the band at ~48 kDa corresponding with amino acids 9–21 of DA D_1_R was detected and quantified [80,81]. For the D_2_R, a pair of bands detected at ~48 and ~51 kDa representing the short and long-forms respectively and corresponding to amino acids 272–282 of DA D_2_R were quantified [82]. For DAT, a band at ~50 kDa was detected and quantified, corresponding to amino acids 541–620 at the C-terminus of the dopamine transporter (DAT) [83]. For TH, a single band at ~58 kDa recognising the C-terminus of TH was detected and quantified [84], whilst for p-TH, a single band at ~60 kDa was detected and quantified [84]. Quantification of the β-actin protein was at 46 kDa. Normalization of results was accomplished by taking the value of the vehicle group as 100%. Each sample for all groups (*n* = 6 per group) has been performed in duplicate to confirm reliability of results.

### 4.5. Receptor Autoradiography and Quantification

Experimental procedures for DAT, D_1_R, and D_2_R binding autoradiography were based on those reported previously [40,85,86,87,88,89].

#### 4.5.1. Dopamine Active Transporter (DAT) Binding Procedures

Brain sections for DAT binding were pre-incubated in 50 mM Tris-HCl buffer containing 120 mM NaCl and 0.1% bovine serum albumin (pH 7.4) for 20 min at 4 °C. Sections were then incubated for 2 h in 15 nM [^3^H]WIN35428 (specific activity, 85.9 Ci/mmol; PerkinElmer, Waltham, MA, USA). Non-specific binding was determined by the addition of 10 µM GBR12909 (Sigma-Aldrich, Castle Hill, NSW, Australia) to subsequent sections. Sections were then washed twice for 1 min in ice-cold buffer, dipped in ice-cold distilled water and then air dried [40,85,88].

#### 4.5.2. Dopamine D_1_ Receptor Binding Procedures

Briefly, brain sections containing PFC, CPu, NAc, Hippocampus, SN and VTA were thawed at room temperature (RT) and pre-incubated in 50 mM Tris-HCl buffer (pH 7.4), containing 120 mM NaCl, 5 mM KCl, 2 mM CaCl_2_ and 1 mM MgCl_2_ for 20 min at RT. Sections were then incubated for 1.5 h at RT in 50 mM Tris-HCl buffer with 4 nM [^3^H]SCH23390 (specific activity: 85 Ci/mmol; PerkinElmer, Waltham, MA, USA) and 30 nM Spiperone (Sigma-Aldrich, Castle Hill, NSW, Australia) to prevent non-specific binding to DA D_2_ receptor. Non-specific binding was determined with the addition of 100 µM (+)-Butaclamol (Sigma-Aldrich, Castle Hill, NSW, Australia) to subsequent sections. Slides with sections were then washed twice for 10 min in ice-cold buffer, dipped in ice-cold distilled water and then dried under a stream of cool air to remove excess buffer salts [40,85].

#### 4.5.3. Dopamine D_2_ Receptor Binding Procedures

D_2_R binding procedures began with pre-incubation for 30 min at RT in 50 mM TrisHCl buffer, containing 120 mM NaCl, 5 mM KCl, 2 mM CaCl_2_, 1 mM MgCl_2_ and 0.001% ascorbic acid (pH 7.4). Incubation for 1 h in 5 nM [^3^H]Raclopride (specific activity 76 Ci/mmol; PerkinElmer, Waltham, MA, USA) at RT was then used for total D_2_ receptor binding. Non-specific binding was determined with addition of 10 µM (+)-Butaclamol (Sigma-Aldrich, Castle Hill, NSW, Australia) to subsequent sections. Following incubation, sections were washed twice for 5 min in ice-cold buffer, dipped in ice-cold distilled water and then air dried [40,85,88,89].

#### 4.5.4. Quantification

All receptor binding slides were exposed to Amersham Hyperfilm ECL (GE Healthcare, Life Science, Wauwatosa, WI, USA) for 2–3 months, along with autoradiographic standards ([^3^H]microscales from Amersham), in X-ray film cassettes. Following exposure time, quantitative analysis of binding images was conducted using the Multi-Analyst image analysis system (Bio-Rad, Hercules, CA, USA), connected to a GS-800 Imaging Densitometer (Bio-Rad, Hercules, CA, USA). Optical density measurement was then converted into fmoles [3H] ligand per mg TE (tissue equivalent) by comparing to the standard. Density of specific binding was calculated through subtraction of non-specific binding from total binding. Confirmation of anatomical structures and specific brain regions was accomplished through reference to a set of Nissl stained (0.5% cresyl violet solution) from each animal, along with a standard rat brain atlas [42].

### 4.6. Statistical Analysis

SPSS software (Windows version 19.0, SPSS Inc., Chicago, IL, USA) was then used to analyse all collected data. Normal distribution of data from all experiments was examined through the use of the Kolmogorov–Smirnov test. All normally distributed data from male and female rats were analysed by two-way analysis of variances (ANOVAs) (Gender × Treatment). Data from males or females were then analysed separately by one-way ANOVA, followed by post hoc Dunnett (correction) tests for multiple comparisons between the treatment groups. Data that were not normally distributed were analysed via the non-parametric Mann–Whitney *U*-test. The data were expressed as mean ± standard error of the mean (SEM). Statistical significance was accepted when *p* < 0.05.

## 5. Conclusions

In summary, this study has revealed that early APD treatment during the critical neurodevelopmental time period has the potential to cause long-lasting alterations to the DA NT system, including changes to DA synthesis markers, transporter and receptor density levels. The observed changes provide potential reasoning for previously documented alterations to behavioural attributes including locomotor activity, cognition and anxiety, previously reported both in our laboratory [31], and other investigations [18,35,37,90], following early treatment with olanzapine, clozapine and risperidone. Although these observed changes to the DA system do provide some insight into and evidence of long-term physiological alterations following early APD use, further investigations into potential effects on other NT groups however may uncover further confounding and more widespread effects related to the previous behavioural alterations observed. Other NT systems including the 5-HT and adrenergic systems are known targets of APD pharmacology, and, furthermore, are well-documented to have significant interplay and thus strong influence on shaping the DAergic signal [56,91,92], as well as a strong correlation to the modulation of anxiety, depressive-like and locomotor behaviours observed in the previously mentioned studies. The modifications uncovered in the present study provides evidence to clinicians of the potential of juvenile treatment with commonly prescribed APDs such as risperidone to result in long-term alterations to the DA NT system in the animal model. The full extent of the potential effects of juvenile APD treatment across multiple brain regions and genders in a clinical setting is currently not well understood, with further investigations (including imaging studies) having the potential to provide further insight into the potential effects of early APD use in children/adolescents, and subsequently allowing clinicians to weigh up the potential risks vs. benefits of APD prescription and use. In particular, the significant alterations to the DA synthesis markers as a result of risperidone treatment in the male cohort should be highlighted, with risperidone currently approved for use in children as young as five in the US, in particular widely used in males.

## Abbreviations

5-HT	Serotonin
ANOVA	Analysis of Variance
APD	Antipsychotic Drug
CPu	Caudate Putamen
DA	Dopamine
DAT	Dopamine Active Transporter
DOPA	3,4-Dihydroxyphenylalanine
HED	Human Equivalent Dose
HRP	Horseradish Peroxidase
NAc	Nucleus Accumbens
NT	Neurotransmitter
p-TH	Phosphorylated-Tyrosine Hydroxylase
PD	Postnatal Day
PFC	Prefrontal Cortex
PVDF	Polyvinylidene Difluoride
RT	Room Temperature
SEM	Standard Error of the Mean
SN	Substantia Nigra
TBST	Tris-Buffered Saline-Tween
TH	Tyrosine Hydroxylase
VTA	Ventral Tegmental Area

## References

[1] Alexander G.C., Gallagher S.A., Mascola A., Moloney R.M., Stafford R.S. (2011). Increasing off-label use of antipsychotic medications in the United States. Pharmacoepidemiol. Drug Saf..

[2] Seida J.C., Schouten J.R., Boylan K., Newton A.S., Mousavi S.S., Beaith A., Vandermeer B., Dryden D.M., Carrey N. (2012). Antipsychotics for children and young adults: A comparative effectiveness review. Pediatrics.

[3] Olfson M., Blanco C., Liu S., Wang S., Correll C.U. (2012). National trends in the office-based treatment of children, adolescents, and adults with antipsychotics. Arch. Gen. Psychiatry.

[4] Rani F., Murray M.L., Byrne P.J., Wong I.C.K. (2008). Epidemiologic features of antipsychotic prescribing to children and adolescents in primary care in the United Kingdom. Pediatrics.

[5] Olfson M., Crystal S., Huang C., Gerhard T. (2010). Trends in antipsychotic drug use by very young, privately insured children. J. Am. Acad. Child Adolesc. Psychiatry.

[6] Varley C.K., McClellan J. (2009). Implications of marked weight gain associated with atypical antipsychotic medications in children and adolescents. JAMA.

[7] Hoekstra P.J. (2014). Risperidone for non-psychotic disorders in paediatric patients: Which child is to benefit?. Dev. Med. Child Neurol..

[8] Andersen S.L., Navalta C.P. (2004). Altering the course of neurodevelopment: A framework for understanding the enduring effects of psychotropic drugs. Int. J. Dev. Neurosci..

[9] Memarzia J., Tracy D., Giaroli G. (2014). The use of antipsychotics in preschoolers: A veto or a sensible last option?. J. Psychopharmacol..

[10] Schneider C., Taylor D., Zalsman G., Frangou S., Kyriakopoulos M. (2014). Antipsychotics use in children and adolescents: An on-going challenge in clinical practice. J. Psychopharmacol..

[11] Haw C., Stubbs J. (2007). Off-label use of antipsychotics: Are we mad?. Expert Opin. Drug Saf..

[12] Vitiello B., Correll C., van Zwieten-Boot B., Zuddas A., Parellada M., Arango C. (2009). Antipsychotics in children and adolescents: Increasing use, evidence for efficacy and safety concerns. Eur. Neuropsychopharmacol..

[13] Sharma A., Shaw S.R. (2012). Efficacy of risperidone in managing maladaptive behaviors for children with autistic spectrum disorder: A meta-analysis. J. Pediatr. Health Care.

[14] Pompili M., Baldessarini R.J., Forte A., Erbuto D., Serafini G., Fiorillo A., Amore M., Girardi P. (2016). Do atypical antipsychotics have antisuicidal effects? A hypothesis-generating overview. Int. J. Mol. Sci..

[15] Grace A.A., Floresco S.B., Goto Y., Lodge D.J. (2007). Regulation of firing of dopaminergic neurons and control of goal-directed behaviors. Trends Neurosci..

[16] Kegeles L.S., Abi-Dargham A., Frankle W.G., Gil R., Cooper T.B., Slifstein M., Hwang D.-R., Huang Y., Haber S.N., Laruelle M. (2010). Increased synaptic dopamine function in associative regions of the striatum in schizophrenia. Arch. Gen. Psychiatry.

[17] Purves-Tyson T.D., Handelsman D.J., Double K.L., Owens S.J., Bustamante S., Weickert C.S. (2012). Testosterone regulation of sex steroid-related mRNAs and dopamine-related mRNAs in adolescent male rat substantia nigra. BMC Neurosci..

[18] Milstein J.A., Elnabawi A., Vinish M., Swanson T., Enos J.K., Bailey A.M., Kolb B., Frost D.O. (2013). Olanzapine treatment of adolescent rats causes enduring specific memory impairments and alters cortical development and function. PLoS ONE.

[19] Meltzer H.Y., Davis K.L., Charney D., Coyle J.T., Nemeroff C. (2002). Mechanism of Action of Atypical Antipsychotic Drugs. Neuropsychopharmacology: The Fifth Generation of Progress.

[20] Nasrallah H.A. (2008). Atypical antipsychotic-induced metabolic side effects: Insights from receptor-binding profiles. Mol. Psychiatry.

[21] Amato D. (2015). Serotonin in antipsychotic drugs action. Behav. Brain Res..

[22] Levitt P., Harvey J.A., Friedman E., Simansky K., Murphy E.H. (1997). New evidence for neurotransmitter influences on brain development. Trends Neurosci..

[23] Frost D.O., Cadet J.L. (2000). Effects of methamphetamine-induced neurotoxicity on the development of neural circuitry: A hypothesis. Behav. Brain Res..

[24] Klomp A., Tremoleda J.L., Wylezinska M., Nederveen A.J., Feenstra M., Gsell W., Reneman L. (2012). Lasting effects of chronic fluoxetine treatment on the late developing rat brain: Age-dependent changes in the serotonergic neurotransmitter system assessed by pharmacological MRI. NeuroImage.

[25] Andersen S.L. (2003). Trajectories of brain development: Point of vulnerability or window of opportunity?. Neurosci. Biobehav. Rev..

[26] Marco E.M., Adriani W., Ruocco L.A., Canese R., Sadile A.G., Laviola G. (2011). Neurobehavioral adaptations to methylphenidate: The issue of early adolescent exposure. Neurosci. Biobehav. Rev..

[27] Cousins L., Goodyer I.M. (2015). Antidepressants and the adolescent brain. J. Psychopharmacol..

[28] Bottelier M.A., Schouw M.L.J., Klomp A., Tamminga H.G.H., Schrantee A.G.M., Bouziane C., de Ruiter M.B., Boer F., Ruhé H.G., Denys D. (2014). The effects of psychotropic drugs on developing brain (ePOD) study: Methods and design. BMC Psychiatry.

[29] Correll C.U. (2010). From receptor pharmacology to improved outcomes: Individualising the selection, dosing, and switching of antipsychotics. Eur. Psychiatry.

[30] Kesby J.P., Cui X., Burne T.H.J., Eyles D.W. (2013). Altered dopamine ontogeny in the developmentally vitamin D deficient rat and its relevance to schizophrenia. Front. Cell. Neurosci..

[31] De Santis M., Lian J., Huang X.F., Deng C. (2016). Early antipsychotic treatment in childhood/adolescent period has long-term effects on depressive-like, anxiety-like and locomotor behaviours in adult rats. J. Psychopharmacol..

[32] Kumra S., Oberstar J.V., Sikich L., Findling R.L., McClellan J.M., Vinogradov S., Schulz S.C. (2008). Efficacy and tolerability of second-generation antipsychotics in children and adolescents with schizophrenia. Schizophr. Bull..

[33] Stigler K.A., McDougle C.J., Posey D.J., Potenza M.N. (2004). Weight gain associated with atypical antipsychotic use in children and adolescents: Prevalence, clinical relevance, and management. Pediatr. Drugs.

[34] Zuddas A., Zanni R., Usala T. (2011). Second generation antipsychotics (SGAs) for non-psychotic disorders in children and adolescents: A review of the randomized controlled studies. Eur. Neuropsychopharmacol..

[35] Shu Q., Hu G., Li M. (2014). Adult response to olanzapine or clozapine treatment is altered by adolescent antipsychotic exposure: A preclinical test in the phencyclidine hyperlocomotion model. J. Psychopharmacol..

[36] Varela F.A., Der-Ghazarian T., Lee R.J., Charntikov S., Crawford C.A., McDougall S.A. (2014). Repeated aripiprazole treatment causes dopamine D_2_ receptor up-regulation and dopamine supersensitivity in young rats. J. Psychopharmacol..

[37] Vinish M., Elnabawi A., Milstein J.A., Burke J.S., Kallevang J.K., Turek K.C., Lansink C.S., Merchenthaler I., Bailey A.M., Kolb B. (2012). Olanzapine treatment of adolescent rats alters adult reward behavior and nucleus accumbens function. Int. J. Neuropsychopharmacol..

[38] Moran-Gates T., Gan L., Park Y.S., Zhang K., Baldessarini R.J., Tarazi F.I. (2006). Repeated antipsychotic drug exposurein developing rats: Dopamine receptor effects. Synapse.

[39] Maciag D., Simpson K.L., Coppinger D., Lu Y., Wang Y., Lin R.C.S., Paul I.A. (2006). Neonatal antidepressant exposure has lasting effects on behavior and serotonin circuitry. Neuropsychopharmacology.

[40] Lian J., Pan B., Deng C. (2016). Early antipsychotic exposure affects serotonin and dopamine receptor binding density differently in selected brain loci of male and female juvenile rats. Pharmacol. Rep..

[41] Frost D.O., Page S.C., Carroll C., Kolb B. (2010). Early exposure to haloperidol or olanzapine induces long-term alterations of dendritic form. Synapse.

[42] Paxinos G., Watson C. (2007). The Rat Brain in Stereotaxic Coordinates.

[43] Der-Ghazarian T., Charntikov S., Varela F., Crawford C., McDougall S. (2010). Effects of repeated and acute aripiprazole or haloperidol treatment on dopamine synthesis in the dorsal striatum of young rats: Comparison to adult rats. J. Neural Transm..

[44] Jordan S., Koprivica V., Dunn R., Tottori K., Kikuchi T., Altar C.A. (2004). In vivo effects of aripiprazole on cortical and striatal dopaminergic and serotonergic function. Eur. J. Pharmacol..

[45] Li Z., Ichikawa J., Dai J., Meltzer H.Y. (2004). Aripiprazole, a novel antipsychotic drug, preferentially increases dopamine release in the prefrontal cortex and hippocampus in rat brain. Eur. J. Pharmacol..

[46] Zocchi A., Fabbri D., Heidbreder C.A. (2005). Aripiprazole increases dopamine but not noradrenaline and serotonin levels in the mouse prefrontal cortex. Neurosci. Lett..

[47] Tanahashi S., Yamamura S., Nakagawa M., Motomura E., Okada M. (2012). Dopamine D_2_ and serotonin 5-H.T._1A_ receptors mediate the actions of aripiprazole in mesocortical and mesoaccumbens transmission. Neuropharmacology.

[48] Etievant A., Betry C., Arnt J., Haddjeri N. (2009). Bifeprunox and aripiprazole suppress in vivo VTA dopaminergic neuronal activity via D_2_ and not D_3_ dopamine autoreceptor activation. Neurosci. Lett..

[49] Verma V., Lim E.P., Han S.P., Nagarajah R., Dawe G.S. (2007). Chronic high-dose haloperidol has qualitatively similar effects to risperidone and clozapine on immediate-early gene and tyrosine hydroxylase expression in the rat locus coeruleus but not medial prefrontal cortex. Neurosci. Res..

[50] Dahan L., Husum H., Mnie-Filali O., Arnt J., Hertel P., Haddjeri N. (2009). Effects of bifeprunox and aripiprazole on rat serotonin and dopamine neuronal activity and anxiolytic behaviour. J. Psychopharmacol..

[51] Tanda G., Valentini V., de Luca M.A., Perra V., Serra G.P., Di Chiara G. (2015). A systematic microdialysis study of dopamine transmission in the accumbens shell/core and prefrontal cortex after acute antipsychotics. Psychopharmacology.

[52] Thompson T.L., Bridges S.R., Weirs W.J. (2001). Alteration of dopamine transport in the striatum and nucleus accumbens of ovariectomized and estrogen-primed rats following *N*-(p-isothiocyanatophenethyl) spiperone (NIPS) treatment. Brain Res. Bull..

[53] Gulley J.M., Zahniser N.R. (2003). Rapid regulation of dopamine transporter function by substrates, blockers and presynaptic receptor ligands. Eur. J. Pharmacol..

[54] Kimmel H.L., Joyce A.R., Carroll F.I., Kuhar M.J. (2001). Dopamine D_1_ and D_2_ receptors influence dopamine transporter synthesis and degradation in the rat. J. Pharmacol. Exp. Ther..

[55] Sinclair D., Purves-Tyson T.D., Allen K.M., Weickert C.S. (2014). Impacts of stress and sex hormones on dopamine neurotransmission in the adolescent brain. Psychopharmacology.

[56] Borgkvist A., Malmlöf T., Feltmann K., Lindskog M., Schilström B. (2012). Dopamine in the hippocampus is cleared by the norepinephrine transporter. Int. J. Neuropsychopharmacol..

[57] Tarazi F.I., Zhang K., Baldessarini R.J. (2001). Long-term effects of olanzapine, risperidone, and quetiapine on dopamine receptor types in regions of rat brain: Implications for antipsychotic drug treatment. J. Pharmacol. Exp. Ther..

[58] Grace A.A. (2000). Gating of information flow within the limbic system and the pathophysiology of schizophrenia. Brain Res. Rev..

[59] Goto Y., Grace A.A. (2005). Dopaminergic modulation of limbic and cortical drive of nucleus accumbens in goal-directed behavior. Nat. Neurosci..

[60] Seeman P. (2008). Dopamine D_2_^High^ receptors moderately elevated by bifeprunox and aripiprazole. Synapse.

[61] Tadokoro S., Okamura N., Sekine Y., Kanahara N., Hashimoto K., Iyo M. (2011). Chronic treatment with aripiprazole prevents development of dopamine supersensitivity and potentially supersensitivity psychosis. Schizophr. Bull..

[62] (2010). Understanding the Risks of Antipsychotic Treatment in Young People. Harv. Ment. Health Lett..

[63] Griffon N., Sokoloff P., Diaz J., Lévesque D., Sautel F., Schwartz J.C., Simon P., Costentin J., Garrido F., Mann A. (1995). The dopamine D_3_ receptor and schizophrenia: Pharmacological, anatomical and genetic approaches. Eur. Neuropsychopharmacol..

[64] Constantine R.J., Boaz T., Tandon R. (2010). Antipsychotic polypharmacy in the treatment of children and adolescents in the fee-for-service component of a large state medicaid program. Clin. Ther..

[65] Purves-Tyson T.D., Owens S.J., Double K.L., Desai R., Handelsman D.J., Weickert C.S. (2014). Testosterone induces molecular changes in dopamine signaling pathway molecules in the adolescent male rat nigrostriatal pathway. PLoS ONE.

[66] Gogos A., Kwek P., van den Buuse M. (2012). The role of estrogen and testosterone in female rats in behavioral models of relevance to schizophrenia. Psychopharmacology.

[67] Dunlop B.W., Nemeroff C.B. (2007). The role of dopamine in the pathophysiology of depression. Arch. Gen. Psychiatry.

[68] Taylor D., Paton C., Kapur S. (2009). The Maudsley Prescribing Guidelines.

[69] Aravagiri M., Marder S.R. (2002). Brain, plasma and tissue pharmacokinetics of risperidone and 9-hydroxyrisperidone after separate oral administration to rats. Psychopharmacology.

[70] Reagan-Shaw S., Nihal M., Ahmad N. (2008). Dose translation from animal to human studies revisited. FASEB J..

[71] Food and Drug Administration (2005). Guidance for Industry: Estimating the Maximum Safe Starting Dose in Initial Clinical Trials for Therapeutics in Adult Healthy Volunteers.

[72] Wadenberg M.L.G. (2007). Bifeprunox: A novel antipsychotic agent with partial agonist properties at dopamine D_2_ and serotonin 5-HT_1A_ receptors. Future Neurol..

[73] Kapur S., VanderSpek S.C., Brownlee B.A., Nobrega J.N. (2003). Antipsychotic dosing in preclinical models is often unrepresentative of the clinical condition: A suggested solution based on in vivo occupancy. J. Pharmacol. Exp. Ther..

[74] Natesan S., Reckless G.E., Nobrega J.N., Fletcher P.J., Kapur S. (2006). Dissociation between in vivo occupancy and functional antagonism of dopamine D_2_ receptors: Comparing aripiprazole to other antipsychotics in animal models. Neuropsychopharmacology.

[75] Lian J., De Santis M., He M., Deng C. (2015). Risperidone-induced weight gain and reduced locomotor activity in juvenile female rats: The role of histaminergic and NPY pathways. Pharmacol. Res..

[76] He M., Zhang Q., Deng C., Wang H., Lian J., Huang X.F. (2014). Hypothalamic histamine H1 receptor-AMP K. signaling time-dependently mediates olanzapine-induced hyperphagia and weight gain in female rats. Psychoneuroendocrinology.

[77] Zhang Q., Lian J., He M., Deng C., Wang H., Huang X.F. (2014). Olanzapine reduced brown adipose tissue thermogenesis and locomotor activity in female rats. Prog. Neuropsychopharmacol. Biol. Psychiatry.

[78] Zhang Q., He M., Deng C., Wang H., Lian J., Huang X.F. (2014). Hypothalamic ghrelin signalling mediates olanzapine-induced hyperphagia and weight gain in female rats. Int. J. Neuropsychopharmacol..

[79] Deng C., Pan B., Hu C.H., Han M., Huang X.F. (2015). Differential effects of short- and long-term antipsychotic treatment on the expression of neuregulin-1 and ErbB4 receptors in the rat brain. Psychiatry Res..

[80] Krishnan B., Centeno M., Pollandt S., Fu Y., Genzer K., Liu J., Gallagher J.P., Shinnick-Gallagher P. (2010). Dopamine receptor mechanisms mediate corticotropin-releasing factor-induced long-term potentiation in the rat amygdala following cocaine withdrawal. Eur. J. Neurosci..

[81] Diaz M.R., Jotty K., Locke J.L., Jones S.R., Valenzuela C.F. (2014). Moderate alcohol exposure during the rat equivalent to the third trimester of human pregnancy alters regulation of GABA_A_ receptor-mediated synaptic transmission by dopamine in the basolateral amygdala. Front. Pediatr..

[82] Karabacak Y., Sase S., Aher Y.D., Sase A., Saroja S.R., Cicvaric A., Höger H., Berger M., Bakulev V., Sitte H. (2015). The effect of modafinil on the rat dopamine transporter and dopamine receptors D_1_–D_3_ paralleling cognitive enhancement in the radial arm maze. Front. Behav. Neurosci..

[83] Pan X., Guo X., Xiong F., Cheng G., Lu Q., Yan H. (2015). Acrylamide increases dopamine levels by affecting dopamine transport and metabolism related genes in the striatal dopaminergic system. Toxicol. Lett..

[84] Pan B., Chen J., Lian J., Huang X.F., Deng C. (2015). Unique effects of acute aripiprazole treatment on the dopamine D_2_ receptor downstream cAMP-PKA and Akt-GSK 3β signalling pathways in rats. PLoS ONE.

[85] Kesby J.P., O’Loan J.C., Alexander S., Deng C., Huang X.F., McGrath J.J., Eyles D.W., Burne T.H.J. (2012). Developmental vitamin D deficiency alters MK-8_01_-induced behaviours in adult offspring. Psychopharmacology.

[86] Lian J., Huang X.F., Pai N., Deng C. (2015). Chronic betahistine co-treatment reverses olanzapine’s effects on dopamine D_2_ but not 5-HT_2A/2C_ bindings in rat brains. Prog. Neuropsychopharmacol. Biol. Psychiatry.

[87] Han M., Huang X.F., Deng C. (2009). Aripiprazole differentially affects mesolimbic and nigrostriatal dopaminergic transmission: Implications for long-term drug efficacy and low extrapyramidal side-effects. Int. J. Neuropsychopharmacol..

[88] du Bois T.M., Hsu C.W., Li Y., Tan Y.Y., Deng C., Huang X.F. (2008). Altered dopamine receptor and dopamine transporter binding and tyrosine hydroxylase mRNA expression following perinatal, NMDA receptor blockade. Neurochem. Res..

[89] Lian J., Huang X.F., Pai N., Deng C. (2013). Effects of olanzapine and betahistine co-treatment on serotonin transporter, 5-HT_2A_ and dopamine D_2_ receptor binding density. Prog. Neuropsychopharmacol. Biol. Psychiatry.

[90] Moe A.A.K., Kurniawan N.D., Alexander S., Cui X., Burne T.H.J., Eyles D.W. (2016). Risperidone induces long-lasting changes in the conditioned avoidance response and accumbal gene expression selectively in animals treated as adolescents. Neuropharmacology.

[91] Guiard B.P., El Mansari M., Blier P. (2008). Cross-talk between dopaminergic and noradrenergic systems in the rat ventral tegmental area, locus ceruleus, and dorsal hippocampus. Mol. Pharmacol..

[92] Kusljic S., Copolov D.L., van den Buuse M. (2003). Differential role of serotonergic projections arising from the dorsal and median raphe nuclei in locomotor hyperactivity and prepulse inhibition. Neuropsychopharmacology.

